# mAb production kinetics in CHO batch culture: exploring extracellular and intracellular dynamics

**DOI:** 10.3389/fbioe.2025.1546105

**Published:** 2025-05-21

**Authors:** Alejandro Avilan Garzon, Bruno Ebel, Cédric Paris, Samuel Schneider, David Pfister, Eric Olmos

**Affiliations:** ^1^ Université de Lorraine, CNRS, LRGP, Nancy, France; ^2^ Ypso-Facto, Nancy, France; ^3^ Laboratoire d’Ingénierie des Biomolécules, LIBio, Université de Lorraine, Nancy, France

**Keywords:** CHO cells, mAb, amino acids, intracellular concentrations, extracellular concentrations

## Abstract

Monoclonal antibodies (mAbs) are complex therapeutic proteins commonly produced by Chinese Hamster Ovary cell culture. Cells are cultivated using a chemically defined medium containing essential nutrients like glucose, amino acids, vitamins, etc., that cells use to grow and produce the target protein among other by-products. Various studies have focused on both extracellular and intracellular culture dynamics, measuring the concentration of various metabolites in the culture medium and inside the cell, but in the vast majority of cases these studies have excluded the intracellular concentration profile of the antibody. To better understand the complexity of the culture process, and the intracellular and extracellular dynamics of the antibody production, the present study focuses on both the extracellular and intracellular biochemical dynamics. A quenching and a lysis protocol were used to obtain the intracellular and the extracellular concentration profiles for the main substrates, metabolites, and mAb during a standard batch culture. The results revealed that three amino acids (glutamine, asparagine, and cystine) were limiting substrates as they were completely depleted almost simultaneously from the culture medium. Intracellular accumulation of different metabolites during the culture process was demonstrated, as well as a 2-day delay between the onset of the intracellular mAb production and its secretion to the culture medium. Finally, a comparison of mass transfer rates across the cell membrane, intracellular production/consumption rates, and accumulation of metabolites in the cell interior revealed that although the intracellular concentrations of the different metabolites changed during the culture process, the dynamics of these variations were much slower than the other two phenomena.

## 1 Introduction

Over the last 30 years, the market for therapeutic proteins produced by the biopharmaceutical industry has been growing steadily, with a projected marked value of $389 billion by 2024 (O’Flaherty et al., 2020; Lagassé et al., 2017). Monoclonal antibodies (mAbs) represent a big part of this market, with an expected market size value of $138.6 billion by 2024 and $300 billion by 2025 (Alejandra et al., 2023). These antibodies are glycoproteins of the immunoglobulin (Ig) family, being the IgG (immunoglobulin G) the most abundant antibody class on the therapeutic antibody market (Lai et al., 2021).

Around 70% of therapeutic recombinant proteins are produced using mammalian cells, with the CHO (Chinese Hamster Ovary) cell line being the preferred expression system, especially for mAb production (O’Flaherty et al., 2020; Frenzel et al., 2013). CHO cells, as well as other mammalian cells require a complex culture medium that contains not only all the required nutriments and proteins to support cell growth but also different molecules that will protect the cells from pH variations and hydrodynamic stresses (Li, 2018). Key components of culture media include glucose as the main source of carbon and energy, glutamine as the main source of nitrogen (Neermann and Wagner, 1996), different amino acids to support protein production (Fan et al., 2015), lipids for cell membrane formation and energy storage (Xu et al., 2005), vitamins in low concentrations used as cofactors (Arora, 2013), and inorganic ions and trace elements with diverse roles such as maintaining the pH and osmolarity of the culture medium (Mizrahi and Lazar, 1988).

Regarding the amino acids, they are the precursors in the synthesis of all proteins in the cell. Twelve of them are considered as “essential” because they cannot be synthesized by the cells (arginine, cysteine, histidine, isoleucine, leucine, lysine, methionine, phenylalanine, threonine, tryptophan, tyrosine, and valine) and therefore are normally added to the culture media (Eagle, 1955; Ritacco et al., 2018). The other amino acids, named ‘non-essential’, can be synthesized by the cell from other precursors present in the culture. These amino acids can also be added to the culture medium to avoid depletion (Li, 2018).

CHO cells present an inefficient energy metabolism characterized by high substrate uptake rates (Pereira et al., 2018). The substrate consumption is not only used for biomass or product formation but between 35% and 70% of glucose can be used in the formation of by-products which, in some cases, can be toxic for the cell. For instance, lactate is a waste product produced in cell culture mostly as a result of glucose metabolism but it can also be produced due to glutamine metabolism (Zielke et al., 1980). Lactate production has a negative effect because at high levels it is toxic and inhibits cell growth by lowering the medium pH (Yilmaz et al., 2020). Above 20 mM, lactate is considered toxic for the cells and has a negative effect on the production and glycosylation of proteins (Lao and Toth, 1997).

One of the major concerns in CHO kinetics studies is the metabolic shift from lactate production to lactate consumption throughout the cell culture. Lactate re-consumption could be considered a desirable phenomenon as it reduces lactate levels in the culture media and thus its undesirable effects (Hartley et al., 2018). For now, the exact mechanism by which this shift arises remains unclear. Different possible explanations made so far includes glucose depletion (Altamirano et al., 2006; Altamirano et al., 2004), glutamine depletion (Ghorbaniaghdam et al., 2014), or a combination of both (Zagari et al., 2013), the extracellular lactate concentration (Kyriakopoulos and Kontoravdi, 2014), a pH shift (Liste-Calleja et al., 2015), or the cell oxidative capacity (Luo et al., 2012).

Ammonium (NH4^+^) is a toxic molecule produced in mammalian cells culture. Ammonium is the product of amino acid metabolism, mainly during the first step of glutaminolysis, when glutamine is converted to glutamate and ammonium ions (Savizi et al., 2021). The inhibitory effect of ammonium accumulation in cell culture media is related to a decrease in available energy in the cell. This is associated with diffusive transport of ammonia through the cell membrane into the abiotic phase, active transport of ammonium through the cell membrane with the help of specific transport proteins, such as Na^+^/K^+^-ATPase, and competition between active transport of ammonium and potassium ions (Yilmaz et al., 2020).

In general, kinetic studies of mammalian cell cultures focus on classic substrates and metabolites such as glucose, glutamine, lactate, and ammonium (Kyriakopoulos et al., 2018). However, different studies have shown that it is also important to monitor the concentrations of some amino acids as they may induce changes in metabolism when they reach certain concentration levels in the culture medium. For example, a study about the effect of asparagine and serine limitation in the culture medium in the case of CHO-GS (Glutamine Synthase) cells found that the absence of asparagine causes cell growth arrest (Duarte et al., 2014). They also found that culture growth was also affected by serine depletion, inducing its metabolic synthesis. Another study confirmed the importance of arginine and other amino acids such as leucine in cell growth and IgG productivity (Fan et al., 2015). They found that the specific amino acid consumption rates are highly dependent on their corresponding abundance in the desired antibody sequence. A different study focused on valine revealed that additions of this amino acid during fed-batch culture reduced ammonia concentration in the culture media, mitigating its negative impact on CHO cell growth (Savizi et al., 2022).

Moreover, other studies have also focused on the intracellular CHO metabolites revealing interesting information such as that cells’ metabolic profile during a perfusion culture, depends on the steady state cell density achieved (Karst et al., 2017), or that peak cell growth is related to a highly glycolytic metabolic state and peak mAb production correspond to a highly oxidative metabolism Templeton et al. (2013). This suggests that intracellular metabolites screening could bring important information that may not be obtained by measuring only extracellular concentrations and that could be used, for example, to improve cell culture kinetic model development. For instance, the concept of accumulation of metabolites inside the cell has been applied recently in the development of kinetic models to predict cell growth and mAb production during fed-batch cell cultures (Ben Yahia et al., 2021).

Focusing on the extracellular concentrations for model development is enough for macroscopic kinetic models that exclude all or most of the details of the intracellular metabolism, linking products to extracellular substrate concentrations, which makes them simple to use and facilitates the determination of model parameters (Ben Yahia et al., 2015; Hagrot et al., 2019). However, the lack of detail of the intracellular dynamics makes accurate predictions difficult when culture conditions are significantly modified (Kyriakopoulos et al., 2018). In contrast, microscopic kinetic models consider the cell metabolism which makes them more biologically relevant but increases model complexity and the associated number of model parameters, thus increasing the need of data required to regress the model parameters (Ben Yahia et al., 2015; Galleguillos et al., 2017; Nolan and Lee, 2011). To overcome the high amount of parameters in microscopic models, some approaches like Flux Balance Analysis (FBA) transform the intracellular metabolites mass balances into an optimization problem, under the hypothesis of a steady state inside the cell (Kyriakopoulos et al., 2018; Orth et al., 2010).

Thus, coupling the screening of extracellular and intracellular metabolites could lead to a better understanding of the complex process of cell culture and the development of more accurate kinetic models. However, measuring both extracellular and intracellular concentrations would require more significant analytical work, making kinetic studies a more time-consuming process. Various studies have included both extracellular and intracellular metabolite screening to understand the lactate metabolic shift (Luo et al., 2012), to design feeding strategies to enhance IgG production (Sellick et al., 2011), or in fluxomic studies to assess the increase in NADPH availability on increased IgG productivity in CHO cells (Wijaya et al., 2021). However, although these studies included various extracellular and intracellular metabolites, they did not include an intracellular IgG concentration profile, which to the best of our knowledge has limited references in the literature (Zhang et al., 2020). In addition, the aforementioned studies do not focus on comparing the rate of accumulation of metabolites inside the cell with the intracellular and extracellular reaction rates during a mAb-producing CHO culture. It would therefore be interesting to compare these phenomena and check whether they are comparable in magnitude or whether one of them predominates over the others. This would indicate whether or not increasing the analytical effort and focusing on both extracellular and intracellular dynamics has a real added value for process modelling and understanding. This comparison would also challenge the hypothesis of a steady state within the cell, as formulated in flux balance analysis.

Thus the aim of the present work is to obtain and compare the extracellular and intracellular kinetic profiles of the IgG, as well as the profiles of the main substrates and metabolites during a CHO batch cell culture, using a quenching and lysis protocol for the measure of the intracellular concentrations. Secondly, the measured concentrations were used to calculate the extracellular and intracellular consumption and production rates of the considered species. Finally, these rates were compared to determine if the accumulation of species inside the cell is significant compared to the transfer across cell membrane and the intracellular reaction rates. This comparison was made using an adaptation of the Thiele modulus used for chemical engineering reactions. While the experiments presented here were conducted in batch culture, the results provide valuable information for designing better feeding strategies in more industrially relevant culture operation modes, such as fed-batch cultures.

## 2 Materials and methods

### 2.1 CHO cells culture

A genetically modified DG44 CHO cell line (CHO M250-9) producing human anti-Rhesus D IgG, was used in this study. The culture medium employed during this work was a serum and protein-free medium mixture consisting of a 1:1 ratio of HyClone™ ActiPro™ (Cytiva, United States) and CD-CHO (Fisher Scientific, United States) supplemented with 4 mM L-glutamine (Sigma Aldrich, United States), and 1% Antibiotic Antimycotic Solution (100 × ) Stabilized (Sigma Aldrich, United States). Cells were cultured during both the preculture and culture in 125 mL Erlenmeyer’s flasks (GRYNIA) with a working volume of 25 mL and were incubated in an agitated incubator (Kuhner, Switzerland) at 37°C, 5% CO_2_, 80% humidity with an agitation rate of 70 rpm.

### 2.2 Sampling and quenching

After the preculture, a total of 11 parallel Erlenmeyer’s flasks were simultaneously seeded at about $3\times 10^{5}$ cell/mL. It was assumed that all the cultures behave the same and only cell density was measured daily to compare them. Each day, an Erlenmeyer’s flask was used to measure the intracellular concentrations following an adaptation of a previously developed quenching and lysis protocol (Kronthaler et al., 2012). During the entire procedure, the sample tubes and all reagents were cooled in ice baths. Once all samples were taken for cell density and extracellular metabolites quantification, the remaining volume of the cell suspension present in the flask was divided into three centrifugation tubes of 50 mL where the cells were rapidly quenched by the addition of five times the volume of precooled phosphate-buffered saline (PBS) (0.5°C) (Sigma Aldrich, United States). The tubes were centrifugated at 1,000 *g* for 1 min at 0°C and the supernatant was discarded. The cell pellets were resuspended in 1 mL of cooled PBS and the centrifugation cycle was repeated. Finally, the cell pellets were frozen in liquid nitrogen and stored at −80°C until metabolite extraction.

### 2.3 Cell lysis-metabolite extraction

#### 2.3.1 Main substrates, metabolites and amino acids extraction

For cell lysis, cell pellets from each centrifugation tube were thawed and resuspended in 1 mL of ice-cold phosphate buffer (10 mM, pH 6.8) and sonicated for 4 min on ice. The solution was then frozen at −80°C for 5 min and then thawed during another sonication process. This procedure was repeated until obtaining three complete freezing/thawing cycles. Samples were centrifugated at 16,233 *g* for 5 min at 4°C. The supernatants were filtered using 0.22 µm PES-Syringe Filters (Dutscher) and stored at −80°C until analysis.

#### 2.3.2 IgG extraction

For IgG intracellular concentrations, a different lysis protocol was used due to the relatively fragile nature of the protein. Cell pellets were thawed and a volume of RIPA™ buffer (Thermo Scientific) was added to obtain a concentration of $50\times 10^{6}$ cell/mL per sample. To increase lysis yield and resuspend cell pellets, samples were sonicated for 30 s with 50% pulse. Samples were then gently shaken for 15 min on ice and finally centrifugated at 14,000 *g* for 15 min. Pellets were discarded and the supernatants were further used for IgG analysis.

### 2.4 Growth and principal metabolites analysis

Viable cell density (VCD), viability, and average diameter of cells were measured daily for each Erlenmeyer’s flask using the Vi-CELL™ cell counter (Beckman Coulter, United States) based on trypan blue dye exclusion of the viable cells. Extracellular and intracellular concentrations of glucose, lactate, glutamine, ammonium ions, IgG, and the lactate dehydrogenase (LDH) (only extracellular) were measured daily for one Erlenmeyer’s flask per day using an automated multiparametric analyzer Gallery™ (Thermo Fisher Scientific, United States). The experiments were performed a total of 3 times (E1, E2, E3) using cells from a different cryotube of the same cell bank each time.

### 2.5 Amino acids analysis

Quantitative analysis of extracellular and intracellular amino acids was realized on a UHPLC-MS system (ThermoFisher Scientific, San Jose, CA, United States) consisting in a quaternary solvent delivery pump connected to a photodiode array detector (PDA) and a LTQ^XL^ mass spectrometer equipped with an atmospheric pressure ionization interface operating in electrospray positive mode (ESI^+^).

Two microliters of sample diluted in water were injected on a C18 column (150 × 2.1 mm, 2.6 µm) (Accucore RP-MS, Thermo Scientific) maintained at 30°C during the run. Mobile phases consisted in water added with 20 mM of nonafluoropentanoic acid (NFPA) for mobile phase A and pure acetonitrile for mobile phase B. The flow rate was set at 0.2 mL/min. Amino acids were eluted using a 12 min linear gradient from 5% to 25% of B followed by a 4 min isocratic step at 25% of B.

Mass spectrometry conditions were as follows: spray voltage was set at 4.5 kV; source gases were set (in arbitrary units/min) for sheath gas, auxiliary gas and sweep gas at 30, 10 and 5, respectively; capillary temperature was set at 300°C; capillary voltage at 5 V; tube lens, split lens and front lens voltages at 45 V, −46 V and −9.5 V, respectively. Ion optics parameters were optimized by automatic tuning using a standard solution of glycine at 0.1 g/L infused in mobile phase (A:B, 1:1) at a flow rate of 5 μL/min. Full scan MS spectra (70–300 m/z) were performed on a LTQ analyzer (Linear Trap Quadrupole). Raw data were processed using the XCALIBUR 2.1 software.

The twenty proteinogenic amino acids (except cysteine replaced by cystine due to the non-reducing conditions implemented) were quantified in extracts thanks to external calibration curves obtained after injection of five standard amino acid solutions from 0.01 to 0.05 mM (these calibration points being injected both at the beginning and at the end of the analytical batch).

As explained above, the experiments were performed in triplicate. However, concerning the intracellular and extracellular concentrations of amino acids obtained by HPLC-MS, the results obtained for the replicate E2 were obviously aberrant and were therefore discarded.

### 2.6 Estimation of intracellular concentrations

For the intracellular concentrations, the collected samples were analyzed using different methods as explained before. The obtained concentrations were then used to calculate the intracellular concentrations ( $\bar{C}$ ) in units of g cell^-1^ L_c_^−1^ or mmol cell^-1^ L_c_^−1^ (where L_c_ is liter of cell volume). For that, the obtained concentrations in the samples were divided by the corresponding cell concentration of the sample using the VCD and cell volume (determined using the cell diameter obtained from the Vi-CELL™ cell counter) of the day.

### 2.7 Determination of growth, death, extracellular consumption and production rates

Cell growth rate (*µ*), death rate (*µ*_*d*_) (both in units of h^-1^), the specific consumption rate (*q*_*s*_), and specific production rate (*q*_*p*_) (both in units of mmol cell^-1^ h^-1^ or g cell^-1^ h^-1^), were calculated from the experimental extracellular data using Equations 1–4.

$$\mu =\frac{1}{VCD}\cdot \frac{d\left(VCD\right)}{dt}$$ (1)

$$\mu_{d}=\frac{1}{VCD}\cdot \frac{d\left(DCD\right)}{dt}$$ (2)

$$q_{s}=\frac{1}{VCD}\cdot \frac{dS}{dt}$$ (3)

$$q_{p}=\frac{1}{VCD}\cdot \frac{dP}{dt}$$ (4)

Where $\frac{d\left(VCD\right)}{dt}$ and $\frac{d\left(DCD\right)}{dt}$ are the time derivatives of the viable cell density and dead cell density respectively (in units of cell L^-1^ h^-1^), and $\frac{dS}{dt}$ and $\frac{dP}{dt}$ are the time derivatives of substrate and product concentration respectively (in units of mmol L^-1^ h^-1^ or g L^-1^ h^-1^). The VCD was used in units of cell/L for the rates determination.

The derivatives of the extracellular experimental data were calculated using different smoothing methods. For day 1 and day 10, the concentration derivatives were calculated using a central difference. For days 2 and 9 a Savitzky-Golay quadratic filter with a window of five was used whereas a window of seven was used for days 3–8. The extracellular concentration derivatives were then divided by the corresponding VCD of the day. For all of the rates, only data from days 1–10 was used.

### 2.8 Determination of intracellular reaction rates

To determine whether the transfer of species across the cell membrane or the intracellular reaction is the limiting step of the culture process, the intracellular reaction rates for the measured substrates, metabolites, and product were calculated. For that, the changes in the extracellular concentrations (*C*) were linked to the fluxes across the cell membrane as shown in Equation 5.

$$\frac{dC}{dt}=\left(F_{\mathrm{out}}-F_{in}\right)\cdot VCD$$ (5)

Where *F*_*in*_ and *F*_*out*_ are the inlet and outlet fluxes from the cell respectively (in units of g cell^-1^ h^-1^ or mmol cell^-1^ h^-1^).

As for the intracellular concentrations, the accumulation or loss of species inside the cell during the culture is due to the mass transfer across the cell membrane as well as the production or consumption reaction for each species, as presented in Equation 6.

$$\frac{d\bar{C}}{dt}=\frac{\left(F_{in}-F_{\mathrm{out}}\right)}{\bar{V_{c}}}-\bar{r}$$ (6)

Where $\bar{V_{c}}$ is the cell volume (in L_c_) and $\bar{r}$ is the intracellular reaction rate (in units of g cell^-1^ L_c_^−1^ h^-1^ or mmol cell^-1^ L_c_^−1^ h^-1^).

Using both Equations 5 and 6, as well as the experimental intracellular and extracellular concentrations, the intracellular reaction rates can be determined.

$$\bar{r}=\frac{d\bar{C}}{dt}+\frac{1}{VCD\cdot \bar{V_{c}}}\cdot \frac{dC}{dt}$$ (7)

Since it is a reaction rate, $\bar{r}$ can be positive or negative depending on whether the intracellular metabolite is produced or consumed, respectively.

Finally, to compare the intracellular reaction rates with the mass transfer across the cell membrane, the following relation was established, inspired by the Thiele modulus used in chemical engineering reactions to relate diffusion and reaction rates:

$$\phi =\frac{\text{reaction rate}}{\text{diffusion rate}}=\frac{\bar{r}}{\bar{r}+\frac{d\bar{C}}{dt}}$$ (8)

If the calculated Thiele modulus is small, the mass transfer rate across the cell membrane is higher than the intracellular reaction rate, and therefore, the accumulation of the species in the cell interior may take place till a critical value that depends on the metabolite considered. On the contrary, if the faster phenomenon is the reaction inside the cell, the Thiele modulus will be high meaning there will not be accumulation of metabolites inside the cell. If the rates of the two phenomena are comparable, the Thiele modulus will be close to 1. This implies that $\frac{d\bar{C}}{dt}$ is negligible compared to $\bar{r}$ and that a quasi-stationary state can be assumed in the intracellular space.

## 3 Results and discussion

**FIGURE 1:**
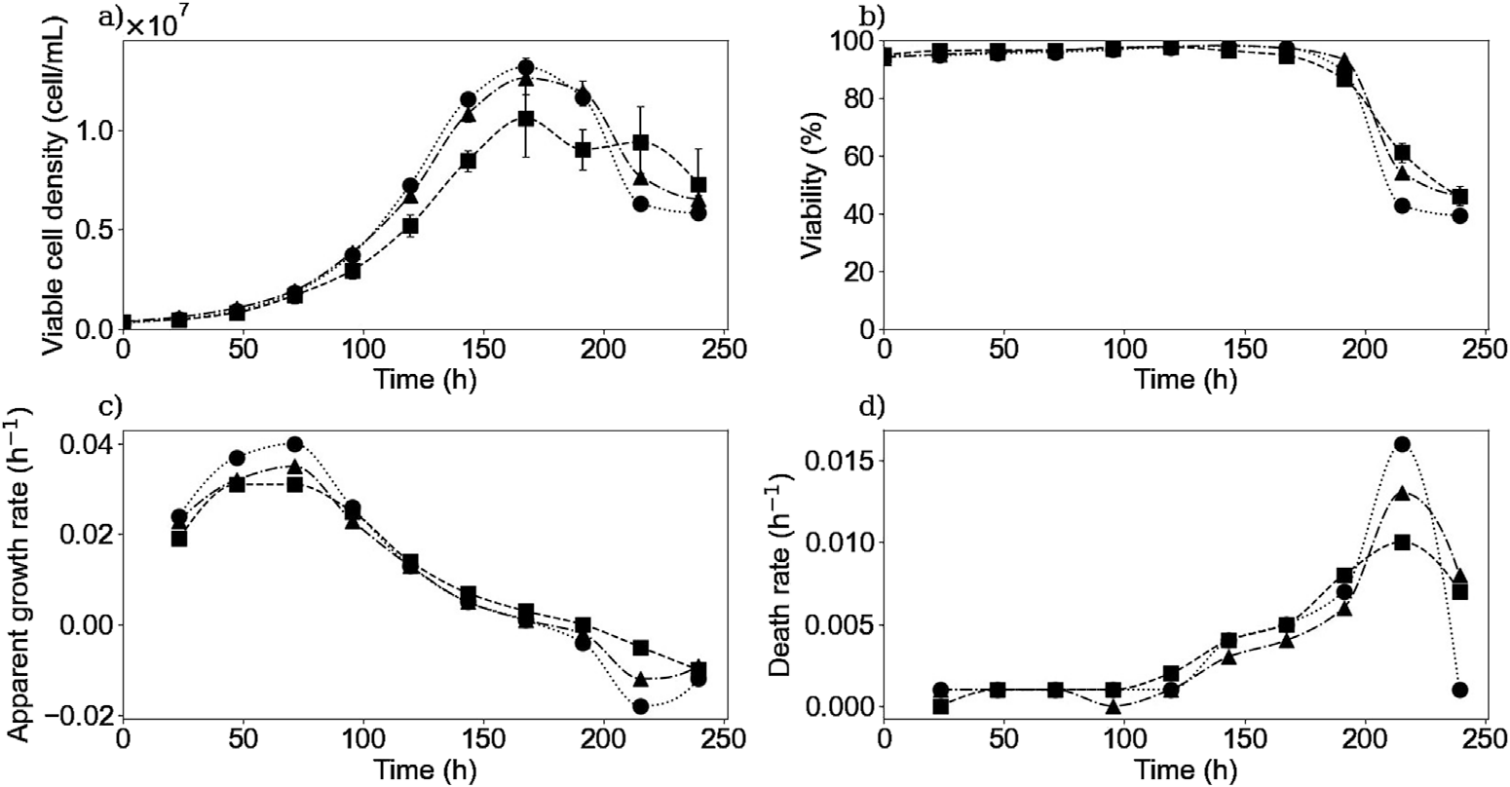
Experimental results of viable cell density **(a)**, cell viability **(b)**, calculated apparent growth rate **(c)**, and death rate **(d)** for experiments E1 (▪), E2 (▲) and E3 (•). Bullets, squares, and triangles represent experimental data or a calculated rate. Error bars represent the standard deviation of the parallel cultures for each repetition.

To study both the extracellular and intracellular dynamics of the IgG production and its link to the different substrates and metabolites present in the culture medium, CHO cell cultures were performed in 11 shaken flasks. These parallel cultures were assumed to behave the same in order to use one of them each day to measure the intracellular and extracellular concentrations of the different substrates, metabolites, and products. Only the viable cell density and the cell viability were measured daily in each of the remaining flasks to verify the culture behavior and the assumption made. Figure 1 shows the experimental VCD and cell viability, as well as the calculated growth rate (Equation 1) for the three repetitions of the performed experiments (E1, E2, and E3). The presented data and the error bars represent the average results and the standard deviation from the measured data from all flasks each day for each repetition. The cultures were performed until day 11 but only data up to day 10 were post-processed; therefore, from this point on, only data from day 0–10 will be presented. The results presented in Figure 1 show that all three replicates remained similar, but most importantly the small error bars in the VCD profiles suggest that the variability remained low and very acceptable within the parallel cultures.

### 3.1 Cell growth kinetics

Concerning the viable cell density profile and the apparent growth rate, they remain similar for the three experiments as seen in Figures 1a,c. Cells from each culture reached on day 7, a maximal cell density of $1.06\times 10^{7}$ , $1.26\times 10^{7}$ and $1.32\times 10^{7}$ cells/mL, and a maximal growth rate of 0.031, 0.035 and 0.040 h^-1^ for experiments E1, E2, and E3 respectively, between the second and third culture day, which is in agreement with previously published results (Zavala-Ortiz et al., 2022). Then, the apparent growth rate progressively decreased during the rest of the experiment until becoming negative by the end of the culture when the global death rate becomes higher than the actual growth rate, as seen in Figure 1d.

### 3.2 Extracellular concentrations, consumption and production rates

#### 3.2.1 Main extracellular substrates and metabolites

**FIGURE 2:**
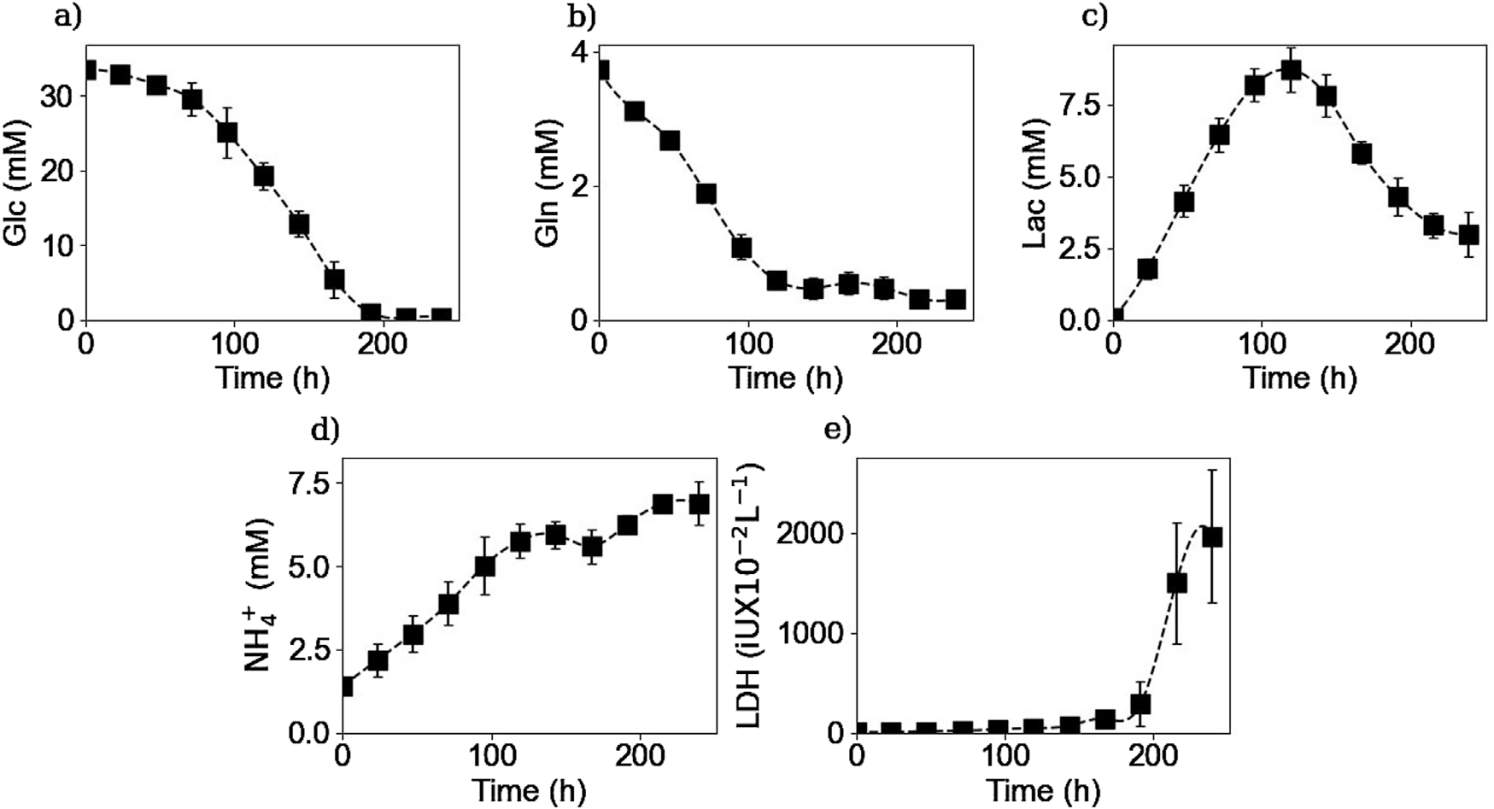
Experimental extracellular concentrations of glucose **(a)**, glutamine **(b)**, lactate **(c)**, ammonium **(d)**, and LDH **(e)**. Squares represent the average of the measured concentrations from the three repetitions (E1, E2, and E3).

**FIGURE 3:**
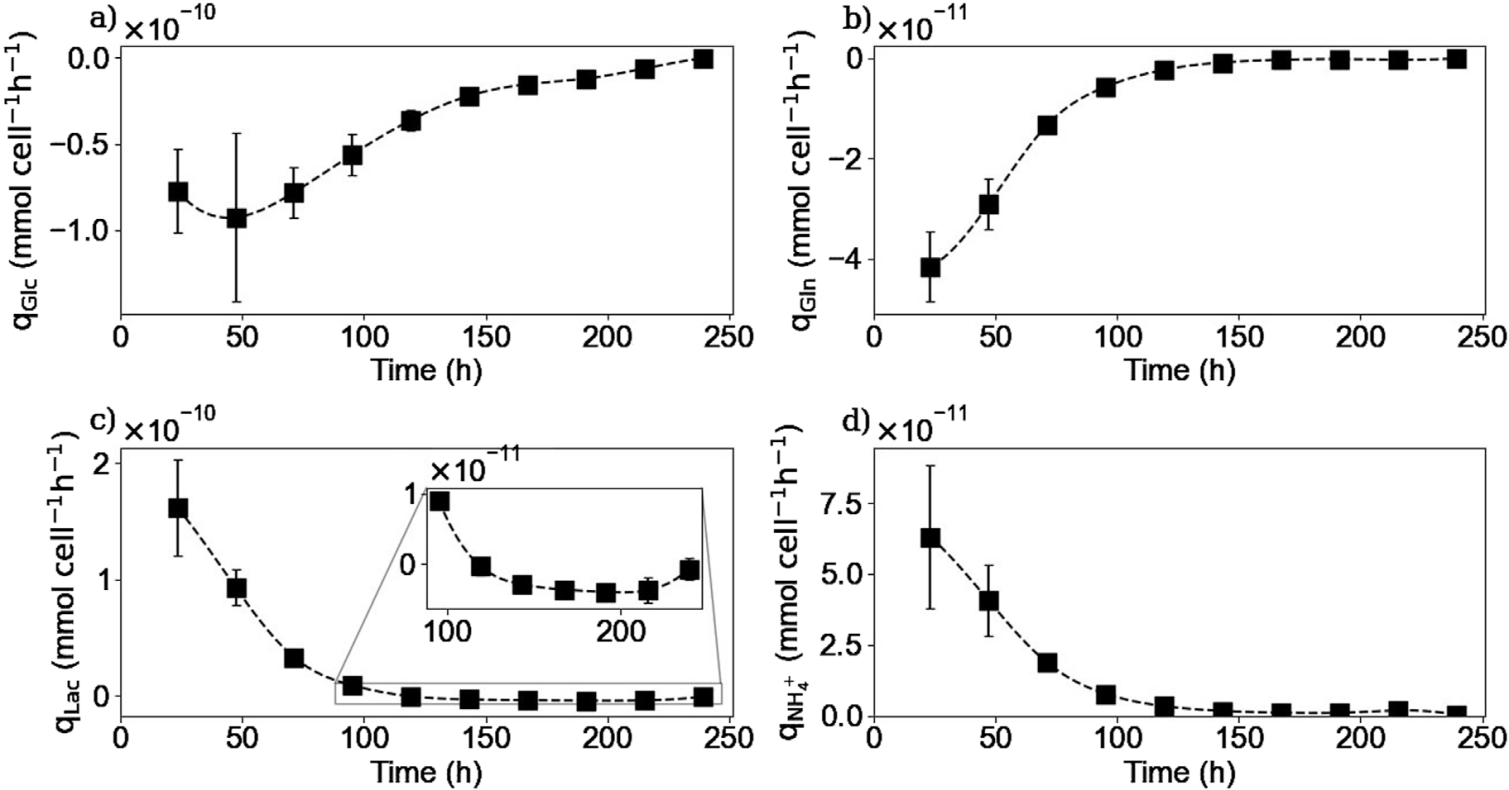
Calculated extracellular specific rates for glucose **(a)**, glutamine **(b)**, lactate **(c)**, and ammonium **(d)**. Squares represent the average of the calculated rates from the three repetitions (E1, E2, and E3).

Figures 2, 3 respectively show the average extracellular concentration kinetics and consumption/production rates for some of the main substrates and metabolites from the three experiments performed. Extracellular glucose (Figure 2a) and glutamine (Figure 2b) were fully consumed after 200 and 150 h of culture respectively, with a maximal consumption rate of $-9.3\times 10^{-11}$ mmol cell^-1^ h^-1^ for glucose and $-4.2\times 10^{-11}$ mmol cell^-1^ h^-1^ for glutamine. In the case of the latter, the measured concentrations by the end of the culture are around 0.5 mM, which falls below the detection limit of the equipment. A concentration of 0 mM was confirmed by HPLC-MS at the end of the culture (data not shown).

Lactate concentration in the culture medium (Figure 2c) increases as the result of glucose metabolism during the first 120 h of the culture, reaching a maximal concentration of 8.6 mM which is far from the toxic lactate concentration of 20 mM reported by Lao et Toth (Lao and Toth, 1997). Afterwards, a shift in lactate production to its consumption is observed during the rest of the culture. This change in lactate metabolism has been previously reported in the literature citing different factors like pH or substrate concentration as possible explanations for this shift, but no consensus has yet been reached (Hartley et al., 2018). During the culture cells presented a maximum lactate production rate of $1.6\times 10^{-10}$ mmol cell^-1^ h^-1^ in the first day, and a maximum consumption rate of $-4.0\times 10^{-12}$ mmol cell^-1^ h^-1^ in the eighth day, when glucose was depleted.

Extracellular NH_4_^+^ (Figure 2d) was produced as expected throughout the culture, with a maximum production rate of $6.3\times 10^{-11}$ mmol cell^-1^ h^-1^, as the result of glutamine and other amino acids metabolization, reaching a high and toxic concentration by the last days of the cultures when cell viability decreased (Lao and Toth, 1997). The calculated consumption and productions rates of glucose, glutamine, lactate, and ammonium are in the same range as preceding values reported in the literature (Zamorano et al., 2010).

Regarding the LDH concentrations, Figure 2e shows that the enzyme concentration increased exponentially after 200 h of culture, consistent with the onset of the decline phase after glucose depletion. This increase in concentration can be linked to an increase in the number of dead cells, since LDH is a non-excreted enzyme so its accumulation in the culture medium is due to the rupture of the cell membrane (Goergen et al., 1993). The LDH measurements were performed to obtain the number of lysed cells as they cannot be measured by the counting method of the Vi-CELL™ cell counter.

#### 3.2.2 Extracellular IgG

**FIGURE 4:**
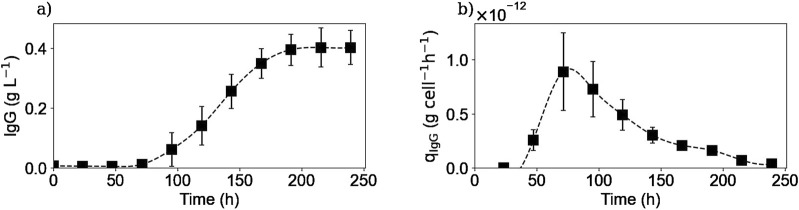
Experimental extracellular IgG concentration **(a)** and calculated extracellular specific IgG production rate **(b)**. Squares represent the average of the experimental concentrations and the calculated rates from the three repetitions (E1, E2, and E3).

Figure 4 shows the extracellular IgG concentration profile and the calculated specific IgG production rate. The antibody extracellular concentration profile (Figure 4a) showed that cells started the IgG secretion after 3 days of culture and reached a plateau of 0.4 g/L after 200 h of culture, with a maximum production rate of $8.9\times 10^{-13}$ g cell^-1^ h^-1^. Thus, the IgG secretion appeared to stop after glucose and other substrates were depleted from the media.

#### 3.2.3 Extracellular amino acids

**FIGURE 5:**
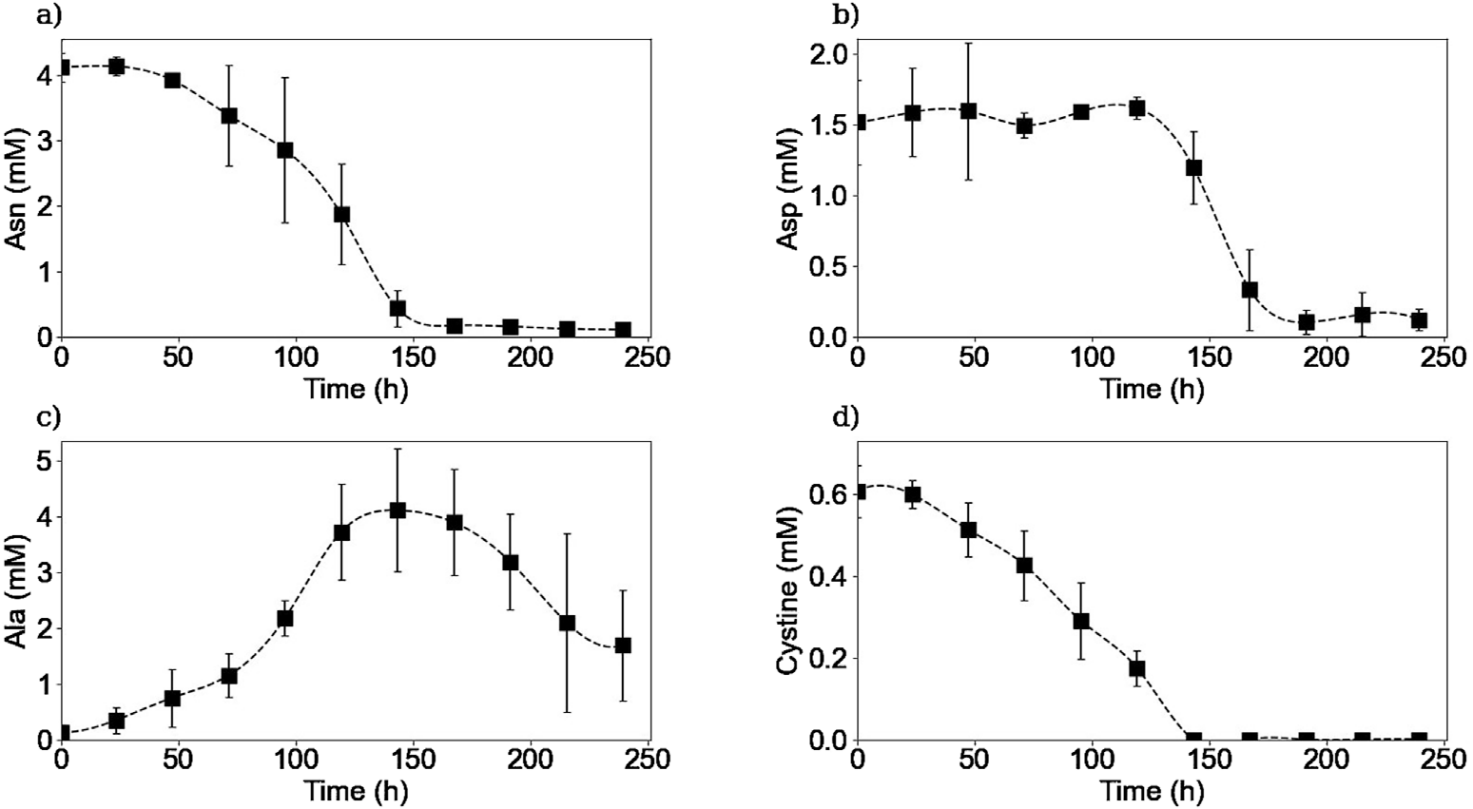
Experimental extracellular concentrations of asparagine **(a)**, aspartic acid **(b)**, alanine **(c)**, and cystine **(d)**. Squares represent the average of the measured concentrations from two repetitions (E1, and E3).

**FIGURE 6:**
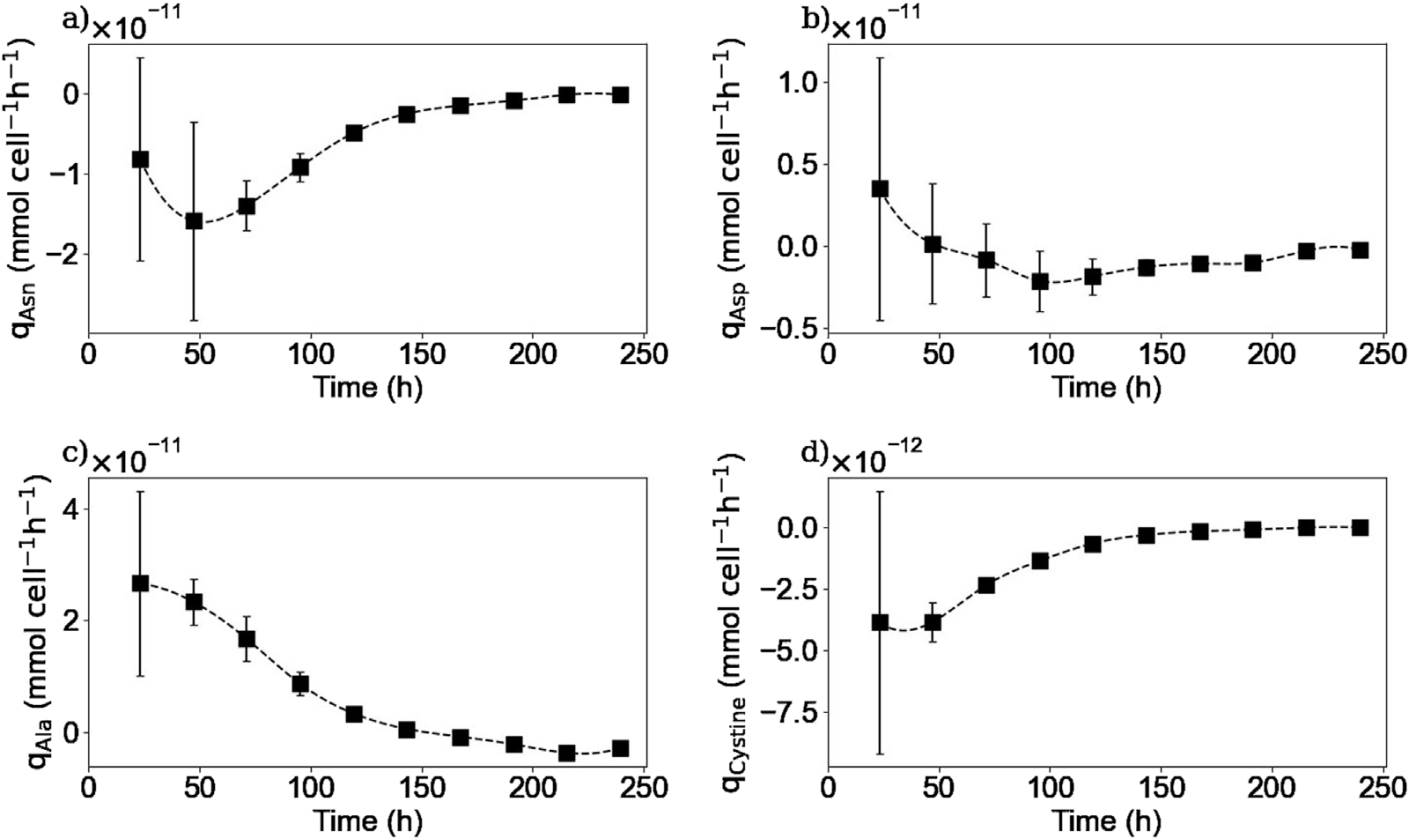
Calculated extracellular specific rates for asparagine **(a)**, aspartic acid **(b)**, alanine **(c)**, cystine **(d)**. Squares represent the average from two repetitions (E1, and E3).

Extracellular amino acid quantification was made using an HPLC-MS method. Figures 5, 6 show the experimental concentrations and the calculated specific consumption or production rates respectively, for selected amino acids. The whole 20 amino acids’ extracellular concentrations kinetics can be found in the Supplementary Material.

As it is shown in Figures 5a, 6a, extracellular asparagine is consumed in the first part of the culture with a maximum specific uptake rate of $-1.6\times 10^{-11}$ mmol cell^-1^ h^-1^ on day 2. Asparagine is completely consumed after 150 h when the culture enters into the stationary phase, which is in agreement with previously published results (Duarte et al., 2014; Carinhas et al., 2013; Wijaya et al., 2021). Asparagine is one of the essential amino acids for CHO cultures and is associated with the production of both alanine and aspartic acid (Yilmaz et al., 2020; Templeton et al., 2013; Vodopivec et al., 2019).

The extracellular concentration of aspartic acid (Figure 5b) remains mostly constant during the first part of the culture and rapidly decreases after 120 h of culture until being almost depleted by 200 h at the end of the stationary phase, in accordance with previous studies (Carinhas et al., 2013). The aspartic acid consumption started when asparagine concentration was halved. One hypothesis to explain this behaviour is that the production of aspartic acid from asparagine consumption was initially sufficient to meet the cells’ needs, and that as soon as the asparagine concentration decreased, the cells began to consume aspartic acid from the culture medium to meet their needs. In other words, this means that asparagine seemed to be preferentially biologically converted to aspartic acid rather than directly take aspartic acid from the culture medium. Another hypothesis could be that cells have a “storage pool” of aspartic acid internally that they use during the first part of the culture. Further investigation is needed in order to confirm this observation. For instance, using radioactive tracers that label the asparagine in the culture medium could be used to determine whether the cell converts asparagine to aspartic acid internally or wheter the aspartic acid used comes from another source.

The extracellular concentration profile of alanine is presented in Figure 5c. As expected for a non-essential amino acid in mammalian cell culture, it is produced in the first part of the culture, reaching a maximum concentration of 4.1 mM at 150 h of culture, with a maximum specific production rate of $2.7\times 10^{-11}$ mmol cell^-1^ h^-1^. The alanine production during the exponential growth phase has already been reported in other studies (Carinhas et al., 2013; Templeton et al., 2013). Then, the alanine metabolism switches from production to consumption from the beginning of the stationary phase until the end of the culture with a maximum specific consumption rate of $-3.6\times 10^{-12}$ mmol cell^-1^ h^-1^. This metabolic switch has been previously reported and related to the asparagine and aspartic acid exhaustion from the culture medium, which is consistent with our experimental results (Carinhas et al., 2013).

Extracellular cystine was measured instead of cysteine due to the spontaneous oxidation of cysteine in aqueous solutions and the non-reducing conditions implemented in the HPLC-MS method. Figure 5d shows that extracellular cystine behaves similarly to other amino acids such as asparagine and glutamine, as it is completely depleted after 150 h of culture. Interestingly, these three substrates were depleted from the media at the same time suggesting that these amino acids are limiting substrates in batch cultures. Cysteine depletion at the beginning of the stationary phase has been reported previously (Sellick et al., 2015). Regarding the other amino acids, as shown in the Supplementary Material, no other amino acids were depleted from the culture medium, but amino acids such as serine, tyrosine, glutamate, tryptophane, and histidine reached minimum levels by the end of the culture, which could also act as limiting substrates. Further experiments should be carried out to determine the effect of these minimum concentrations of the different amino acids on the cell culture.

### 3.3 Intracellular concentrations, consumption and production rates

To test the effectiveness of the cell lysis protocol, a cell count using the Vi-CELL™ cell counter was performed in a cell culture sample before and after the protocol application. The results confirmed that the sonication/refrigeration cycles were able to lyse all the cells in the sample so that the measured metabolite concentration correctly corresponded to the combined content of all cells. An image of the cells before and after the lysis protocol can be found in the Supplementary Material.

#### 3.3.1 Main intracellular substrates and metabolites

**FIGURE 7:**
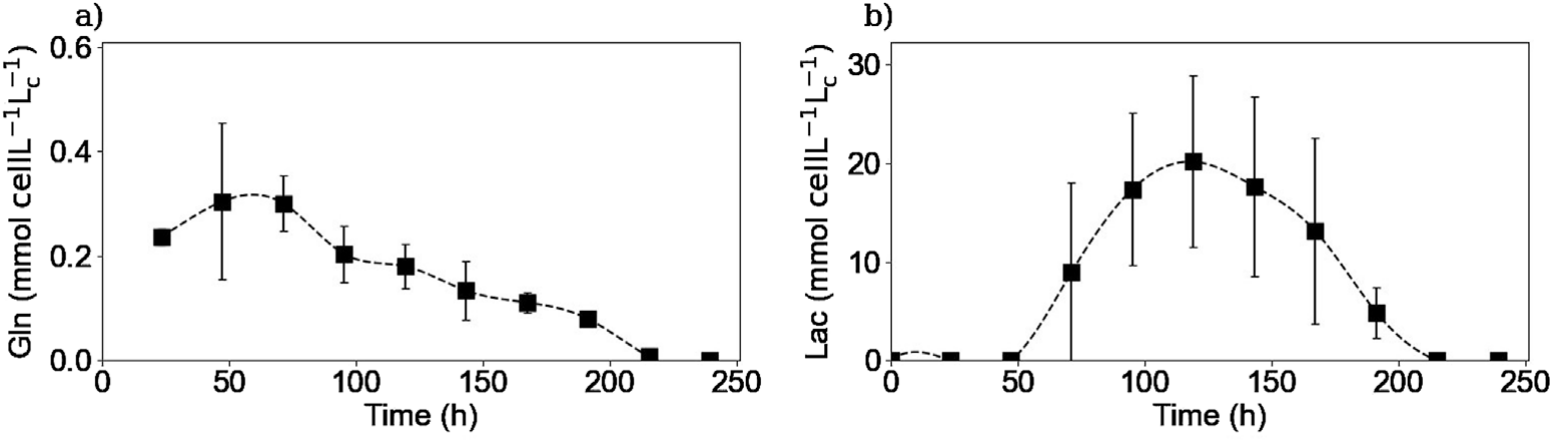
Experimental intracellular concentrations of glutamine **(a)**, and lactate **(b)**. Glutamine concentrations were measured via HPLC-MS. Lactate concentrations were obtained using the Gallery™ equipment. Squares represent the average of the measured concentrations from experiments E1 and E3 for glutamine and for the three repetitions (E1, E2, and E3) for lactate.

Figure 7 shows the intracellular concentrations of selected metabolites. For glucose, glutamine, and NH4^+^ measurements, the concentrations obtained were within the limit of detection of the method because the samples were highly diluted, and therefore no definitive conclusions could be drawn. For glutamine as for the other amino acids, the intracellular concentrations were also measured by HPLC-MS, which has a higher sensitivity. Figure 7a shows that intracellular glutamine concentration starts increasing in the first 2 days of the culture. Then, it decreases and after 200 h, no more glutamine is detected inside the cell. It is interesting to note that even after 150 h of culture when there was no more extracellular glutamine, there was still some glutamine left inside the cell. The cells could use this glutamine accumulation to prolong their metabolic activity for a few more hours of culture.

Figure 7b shows that intracellular lactate began to accumulate until it reached a maximum between 100 and 120 h and then decreased during the remainder of the culture. This coincided with the time when the lactate metabolic shift occurred and continued until no more lactate was measured inside the cell, although extracellular lactate was available for consumption.

#### 3.3.2 Intracellular IgG

**FIGURE 8:**
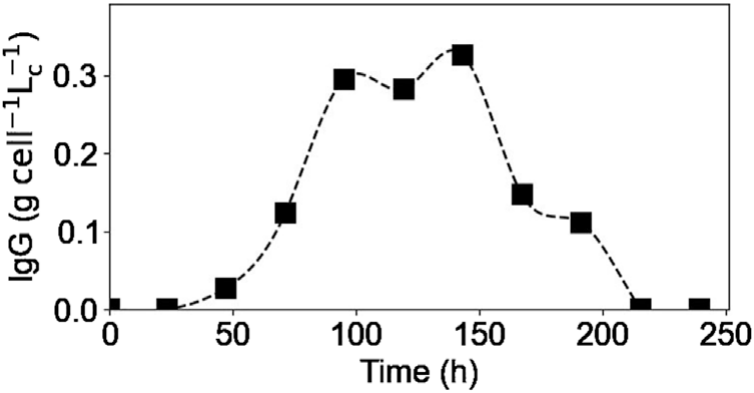
Experimental intracellular IgG concentrations. Squares represent the measured concentrations from experiment E4.

Figure 8 shows the concentrations obtained using the second lysis protocol for the intracellular IgG concentration. A new culture was carried out for this test (E4), using the same culture conditions and obtaining the same extracellular profiles as in the previous experiments (data not shown). As it can be seen, the intracellular IgG concentration increased at the beginning of the culture, reaching a plateau between 100 and 150 h of culture and, finally decreased until reaching a value of 0 after 200 h of culture. The shape of this intracellular concentration profile is similar to that published in the Supplementary Material of another study (Zhang et al., 2020). When compared to the extracellular IgG profile (Figure 4a), it is interesting to see that the extracellular concentration started to increase almost 48 h after the intracellular concentration did, meaning that there is a 2-day delay between the two profiles. This difference could be due to several factors, for example, the cell may need to accumulate a certain amount of antibodies before excreting them, or the antibodies measured inside the cell may not be fully formed and matured, and therefore need more time to complete the process before being excreted. Further investigation is here needed to validate these preliminary hypotheses. Moreover, this difference between extracellular and intracellular concentration profiles may provide clues to improve models for controlling and optimizing monoclonal antibody production processes. In fact, focusing only on the extracellular concentration would not take into account that intracellular production has already been completed days before.

In addition, the end of the intracellular IgG plateau matches the moment when different amino acids (i.e., glutamine, asparagine, cystine) were fully depleted from the culture medium meaning that cells’ antibody production was limited to an intracellular amino acids stock. Therefore, the decrease in the intracellular IgG concentration corresponds to a lower intracellular production rate compared to the IgG secretion rate. This could explain why the extracellular IgG concentration continued to increase, at a much slower rate, even after some of the amino acids were no longer present in the culture media and reached a plateau after 200 h of culture, when there were no more antibody inside the cells. This concept of accumulation of metabolites inside the cell to account for the product synthesis, even if a substrate has been depleted from the culture medium, has been previously reported (Ben Yahia et al., 2021), and is thus demonstrated in the present study. To improve the IgG production rate, it would be thus interesting to study the intracellular IgG concentration profile during a fed-batch culture; this would allow to verify whether the addition of the depleted amino acids would modify the concentration plateau obtained, reaching a higher intracellular concentration or prolonging it.

The intracellular lactate and IgG concentration profiles appeared to be very similar. However, based on the data obtained, it was not possible to suggest a possible biological connection between them.

#### 3.3.3 Intracellular amino acids

Due to the small sample volume collected for day 0, it was not possible to measure the intracellular amino acid concentrations that day; therefore only data from days 1–10 are presented. The full 20 amino acid intracellular concentration kinetics can be found in the Supplementary Material.

**FIGURE 9:**
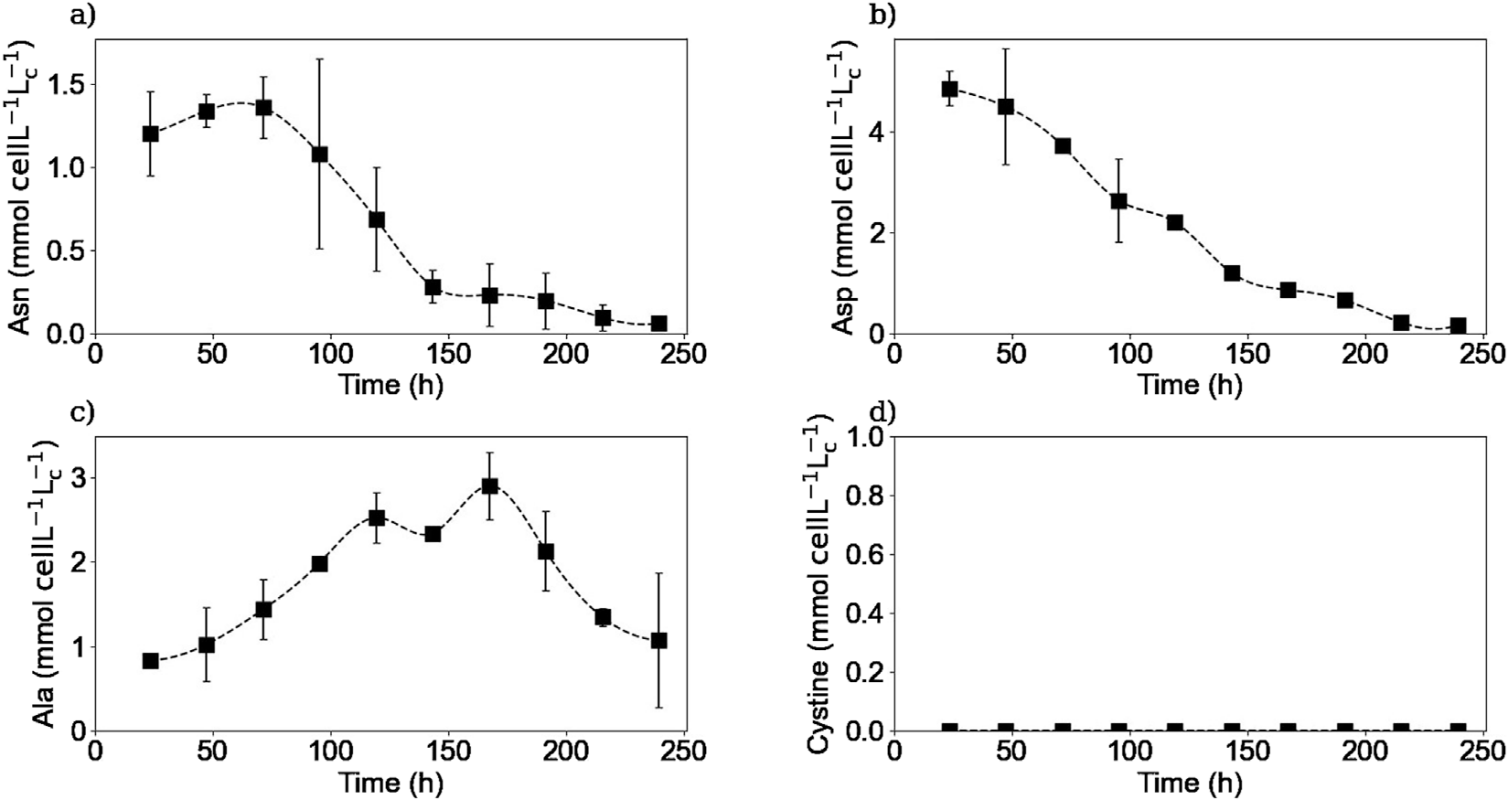
Experimental intracellular concentrations of asparagine **(a)**, aspartic acid **(b)**, alanine **(c)**, and cystine **(d)**. Squares represent the average of the measured concentrations from two repetitions (E1 and E3).

Figure 9 shows the experimental intracellular concentrations for asparagine, aspartic acid, alanine, and cystine. Intracellular asparagine concentration profile is shown in Figure 9a. As it can be seen, the asparagine intracellular concentration increased during the first 3 days, and then, during the rest of the culture, the intracellular concentration decreased. This increase in concentration during the early exponential phase and decrease during the exponential and decline phases has been previously reported in different studies (Templeton et al., 2013; Vodopivec et al., 2019; Dean and Reddy, 2013).

Figure 9b shows aspartic acid intracellular concentration profile. As it can be seen, the aspartic acid is consumed in the cell interior during all the stages of the culture. The decrease in intracellular aspartic acid concentration during the decline phase has been reported in another study (Vodopivec et al., 2019). This continuous decrease in the intracellular concentration confirms that aspartic acid was constantly being metabolized even though the extracellular aspartic acid concentration remained fairly constant during the first part of the culture.

Moreover, during the exponential phase, the intracellular alanine concentration increased as a result of its synthesis in the cell interior (Figure 9c) (Coulet et al., 2022). Then, during the decline phase, the intracellular alanine was consumed following the alanine metabolic switch, leading to the decrease of the extracellular alanine concentration. Concerning intracellular cystine concentrations, Figure 9d shows that, at least with the presented experimental protocol and analytical methods, there was no accumulation of cystine, in the form of molecular cystine, in the cell interior. As explained before, cystine was measured instead of cysteine due to the spontaneous oxidation of cysteine in aqueous solutions and the non-reducing conditions implemented in the HPLC-MS method. The equipment’s detection limit for cystine was 0.01 mM.

These data on the intracellular concentration of amino acids could be complemented in a future study by measurements of intracellular enzymes, possibly linking the profiles obtained to mechanisms of allosteric enzyme regulation (O’Brien et al., 2020; Dean and Reddy, 2013).

#### 3.3.4 Intracellular reaction rates

Using both the extracellular and intracellular concentrations, the intracellular reaction rates for different species were calculated using Equation 7. Due to the uncertainty of the glucose and ammonium concentration results inside the cell, the reaction rates were not calculated for these two species. Also, because intracellular concentrations were not measured on day 0 for HPLC-MS analysis, the reaction rates of the amino acids were calculated from day 2.

**FIGURE 10:**
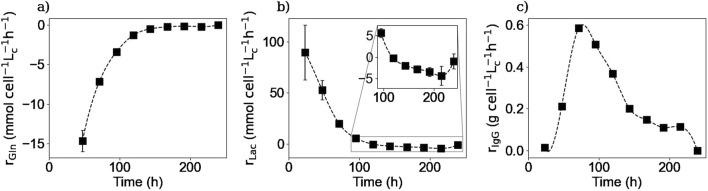
Calculated intracellular reaction rates for glutamine **(a)**, lactate **(b)**, and IgG **(c)**. Squares represent the average of the calculated reaction rates from repetitions E1 and E3 for glutamine, E1, E2 and E3 for lactate and E4 for the IgG.

**FIGURE 11:**
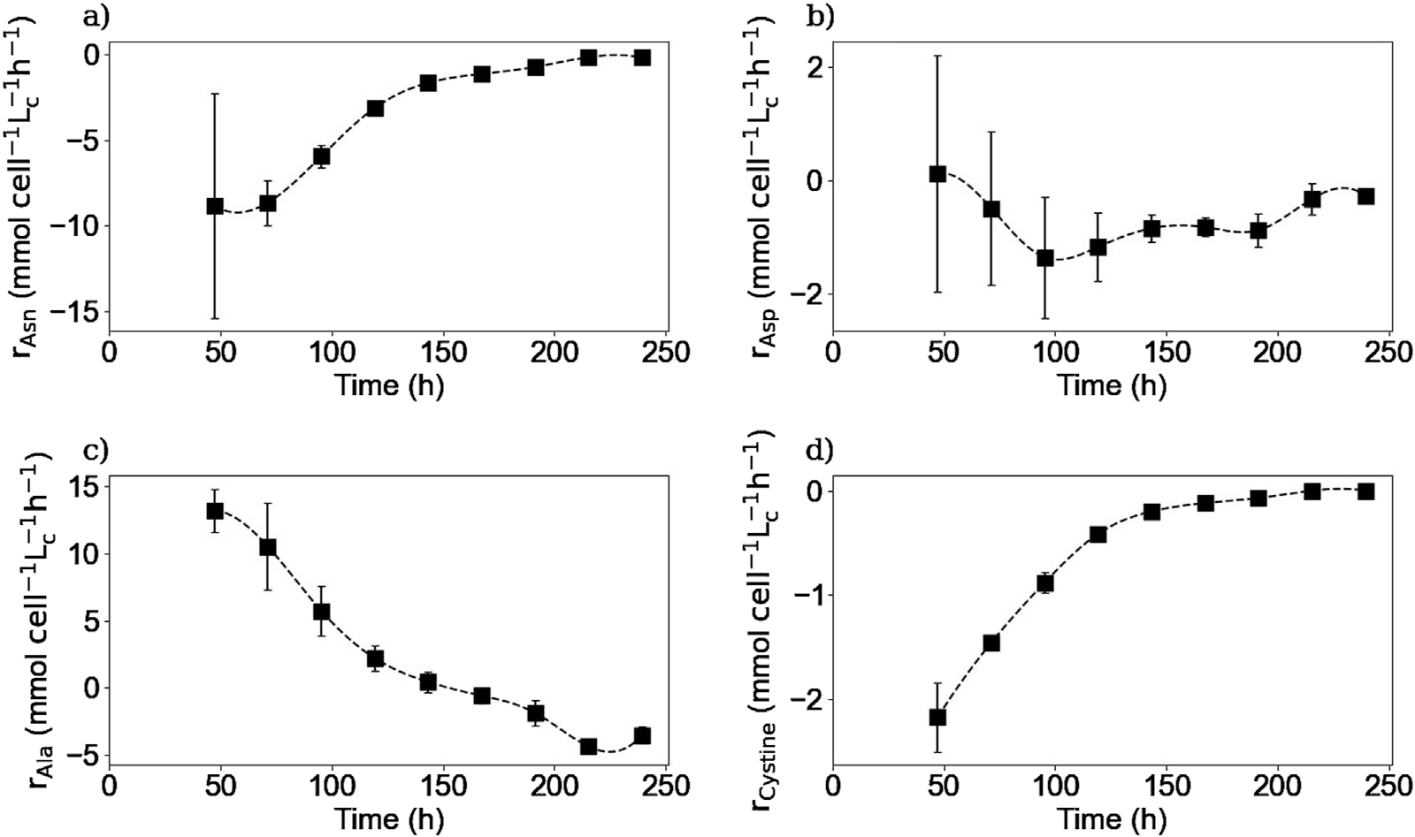
Calculated intracellular reaction rates for asparagine **(a)**, aspartic acid **(b)**, alanine **(c)**, and cystine **(d)**. Squares represent the average of the calculated reaction rates from repetitions E1 and E3.

Figures 10, 11 show the intracellular reaction rates for selected species. The reaction rates for all of the twenty amino acids are given in the Supplementary Material. The reaction rates were calculated as production rates therefore if the rate was positive, the metabolite was produced and if it was negative, it was consumed. As it can be seen in Figures 10a, 11a, the intracellular reaction rates for glutamine and asparagine, two of the amino acids consumed, were maximum on day 2 for glutamine and between days 2 and 3 for asparagine, and then decreased as the intracellular concentration of both species also decreased. In the case of aspartic acid, the maximal reaction rate was on day 4 (Figure 11b). The larger errors observed between days 2 and 4 were due to slightly different values of aspartic acid concentrations between these two times. These errors were amplified by the derivative post-processing of the data.

Intracellular reaction rates for lactate and alanine are presented in Figures 10b, 11c. Both molecules were produced at the beginning of the culture and presented a maximum production rate on day 1 for lactate and day 2 for alanine. Then, as explained before, both metabolites had a metabolic switch in the last part of the culture, and therefore, the calculated intracellular reaction rates became negative during the same period. In the case of cystine, the calculated intracellular reaction rates profile perfectly matched the specific consumption rate profile (Figure 11d); the concentrations obtained inside the cell were indeed close to zero, indicating there was no accumuation of this amino acid in this form inside the cell. Concerning the intracellular reaction rates of IgG, its maximal intracellular production rate was obtained during day 3 and then decreased during the rest of the culture (Figure 10c).

For lactate, IgG, asparagine, aspartic acid, and cystine, the days on which the maximum intracellular reaction rates were obtained, coincided with the days on which the specific consumption or production rates for these molecules were calculated. These results are particularly interesting for the IgG kinetics, because even with the 2-day delay between the intracellular and extracellular antibody concentrations, the peaks of both rates matched, suggesting that the intracellular concentrations are negligible compared to both rates. In the case of alanine and glutamine, the maximum specific production and consumption rates, were obtained on day 1. However, since the intracellular reaction rates could not be calculated on day 1, it was not possible to verify whether the peaks corresponded to the same day.

Finally, the measured extracellular and intracellular concentrations as well as the calculated rates were used to compare the intracellular and extracellular dynamics. To that end, the mass transfer across the cell membrane was compared with the intracellular reaction rates for each substrate, metabolite, and product, using the adaptation of the Thiele modulus presented in Equation 8. A value very close to one was obtained for all the species during the whole culture, meaning that both phenomena are comparable. Therefore, although variations in intracellular metabolite concentrations were observed experimentally, the dynamics of these variations were largely slower than the mass transfer across the cell membrane or than intracellular reaction kinetics. These results are in agreement with the hypothesis of a pseudo-steady state inside the cell made in some culture modeling approaches such as the FBA. This does not mean that the intracellular concentrations do not change during the culture duration but that, these changes can be neglected on a short time scale compared to the changes in extracellular concentrations. Furthermore, this pseudo-steady state implies that the extracellular and intracellular dynamics do not have the same time scale, as the changes inside the cells occurs much quicker than the changes in the extracellular environment and therefore, focusing only on extracellular dynamics would overlook all the intracellular dynamics and all the information it can provide to model development and understanding of the culture process.

## 4 Conclusion

The present study focused on the intracellular and extracellular dynamics during a mAb-producing CHO batch culture. Concerning the extracellular metabolites concentration, it was shown that different amino acids like glutamine, asparagine, and cystine were completely depleted from the culture media at the same time, when the culture entered the stationary phase, and cell growth stopped, suggesting that those amino acids are limiting substrates during batch cultures for the used cell line.

As for the intracellular concentrations, different metabolites were measured and their intracellular concentration profiles were obtained. It was confirmed that the concentrations of the different metabolites inside the cell do not remain constant, but change during the culture. Cells seem to keep a storage of metabolites, such as amino acids (e.g., glutamine and asparagine), for their use, allowing the cells to continue their metabolic activity for some time after these metabolites have been completely depleted from the culture medium. Extracellular metabolites that underwent a metabolic switch from production to consumption, such as lactate and alanine, also reflected this change in the intracellular concentration profiles. In the case of the intracellular IgG concentration, it was found that there was a 2-day delay between intracellular antibody production and its secretion to the culture medium. This finding may provide clues to improve models for controlling and optimizing monoclonal antibody production processes, as focusing only on the extracellular concentration would not take into account that intracellular production has already been completed days before.

The profiles of extracellular and intracellular concentrations showed that, although the concentrations inside the cell do not remain constant, compared to the mass transfer rate across the membrane and the reaction rate inside the cell, the rate of intracellular accumulation is negligible, confirming a pseudo-steady state inside the cell during a short time period, and thus validating the assumptions made in modeling approaches such as the FBA. Finally, it was shown that focusing on both the extracellular and intracellular dynamics could improve process understanding and kinetic model development as both dynamics bring important information about different phenomena that occur at different time scales.

## Data Availability

The raw data supporting the conclusions of this article will be made available by the authors, without undue reservation.
