# Supplementary material for: mAb production kinetics in CHO batch culture: exploring extracellular and intracellular dynamics
Source: Front Bioeng Biotechnol. 2025 May 21;13:1546105. doi: 10.3389/fbioe.2025.1546105 (PMC12134900; doi:10.3389/fbioe.2025.1546105)
Supplement: Supplementary file 1 [file DataSheet1.docx]

**Supplementary data**


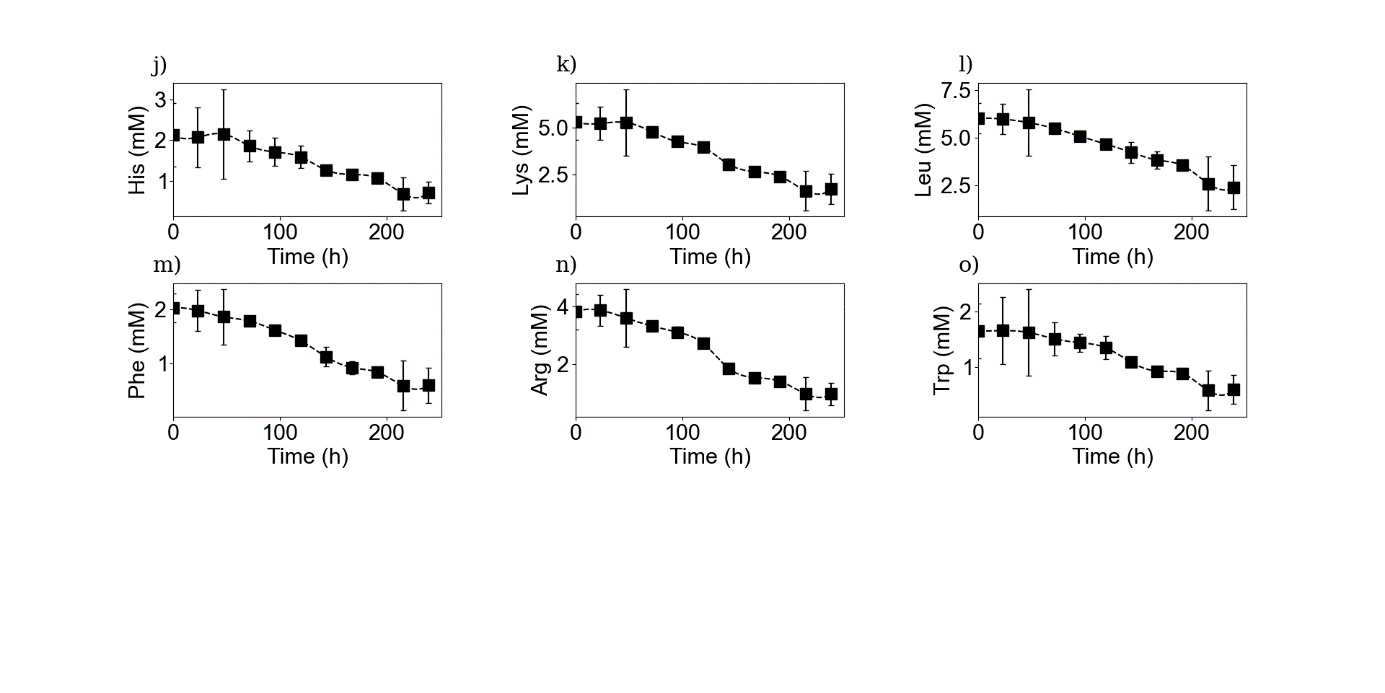

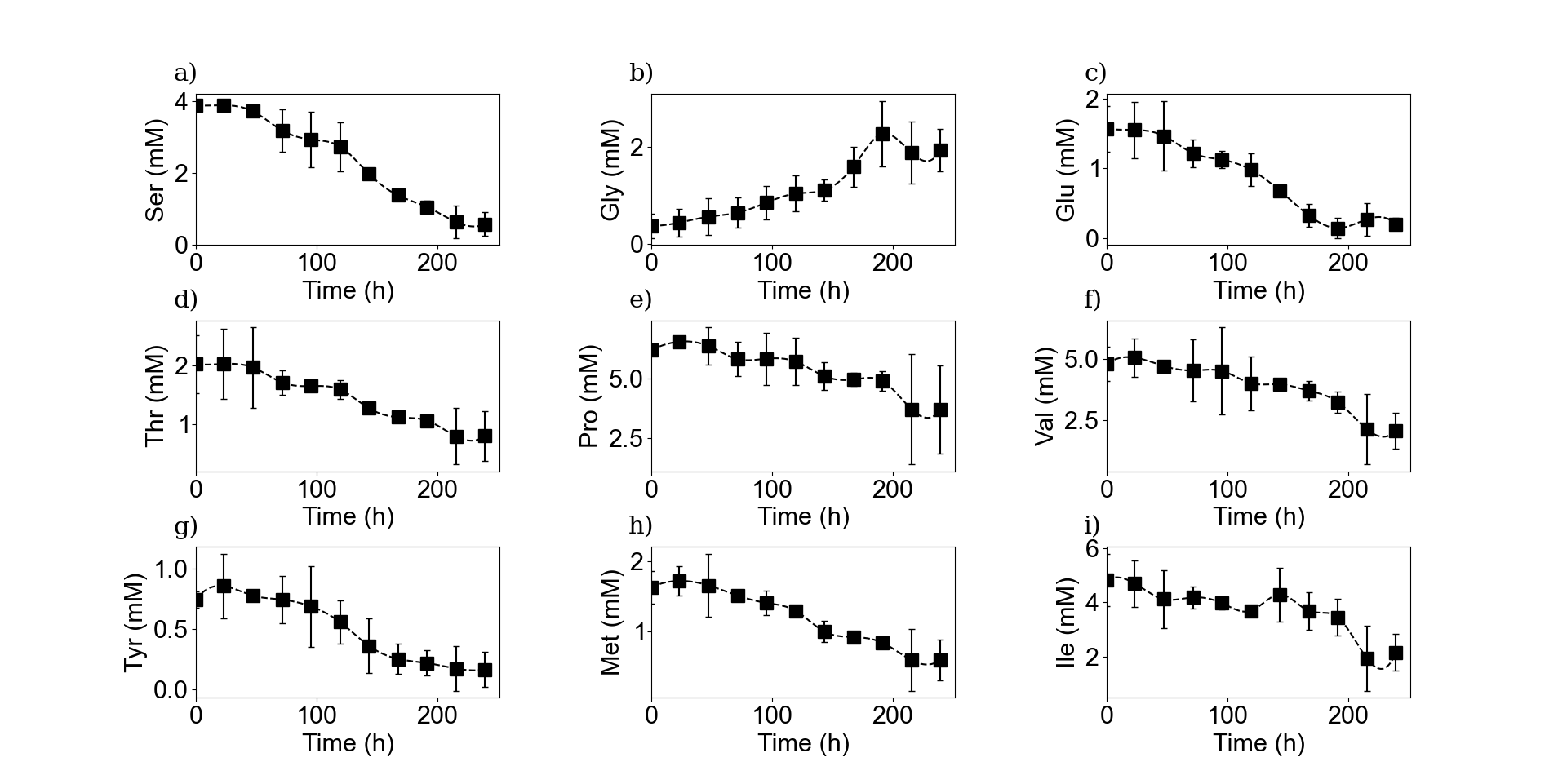


**Figure S 1.** Experimental extracellular amino acids concentrations. Squares represent the average of the measured concentrations from 2 repetitions (E1 and E3).

**Figure S 2.** Calculated extracellular amino acids specific rates. Squares represent the average from 2 repetitions (E1 and E3).


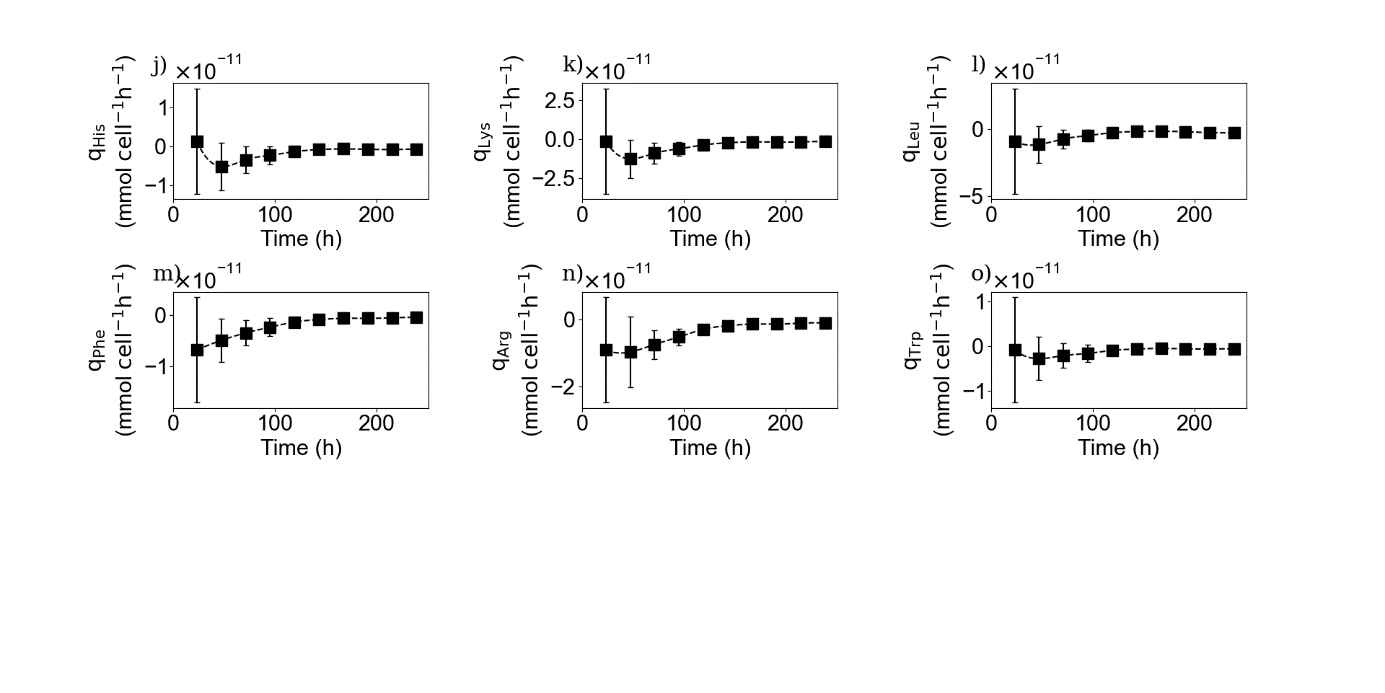

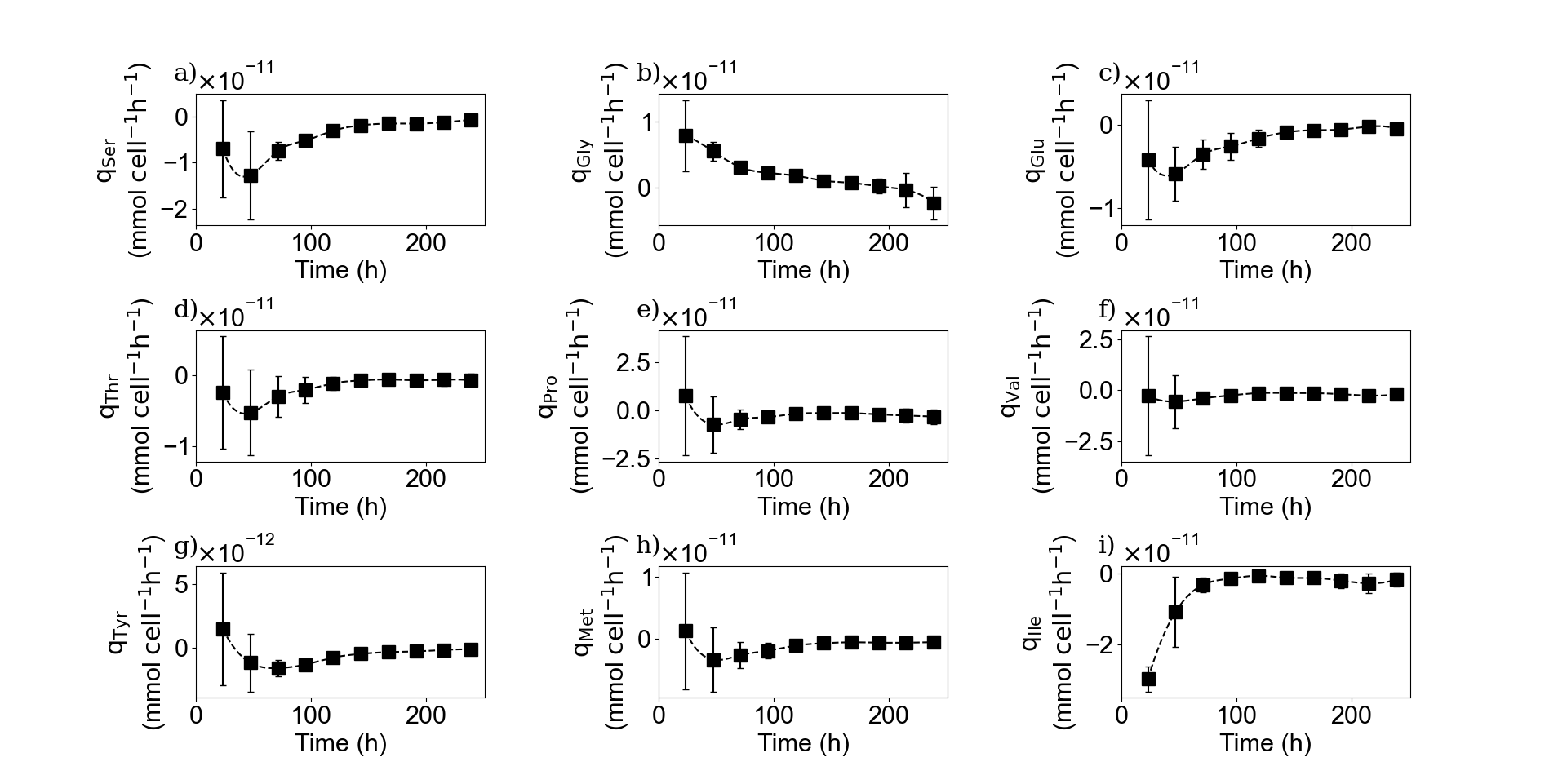


**Figure S 3**. Comparison of cell count using the Vi-CELL ^TM^ cell counter before (left), and after (right) the lysis protocol.


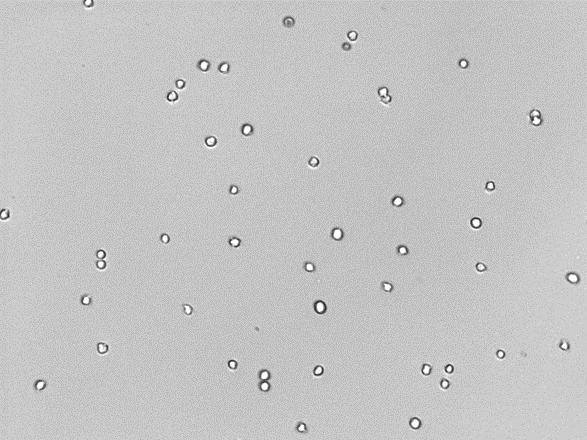

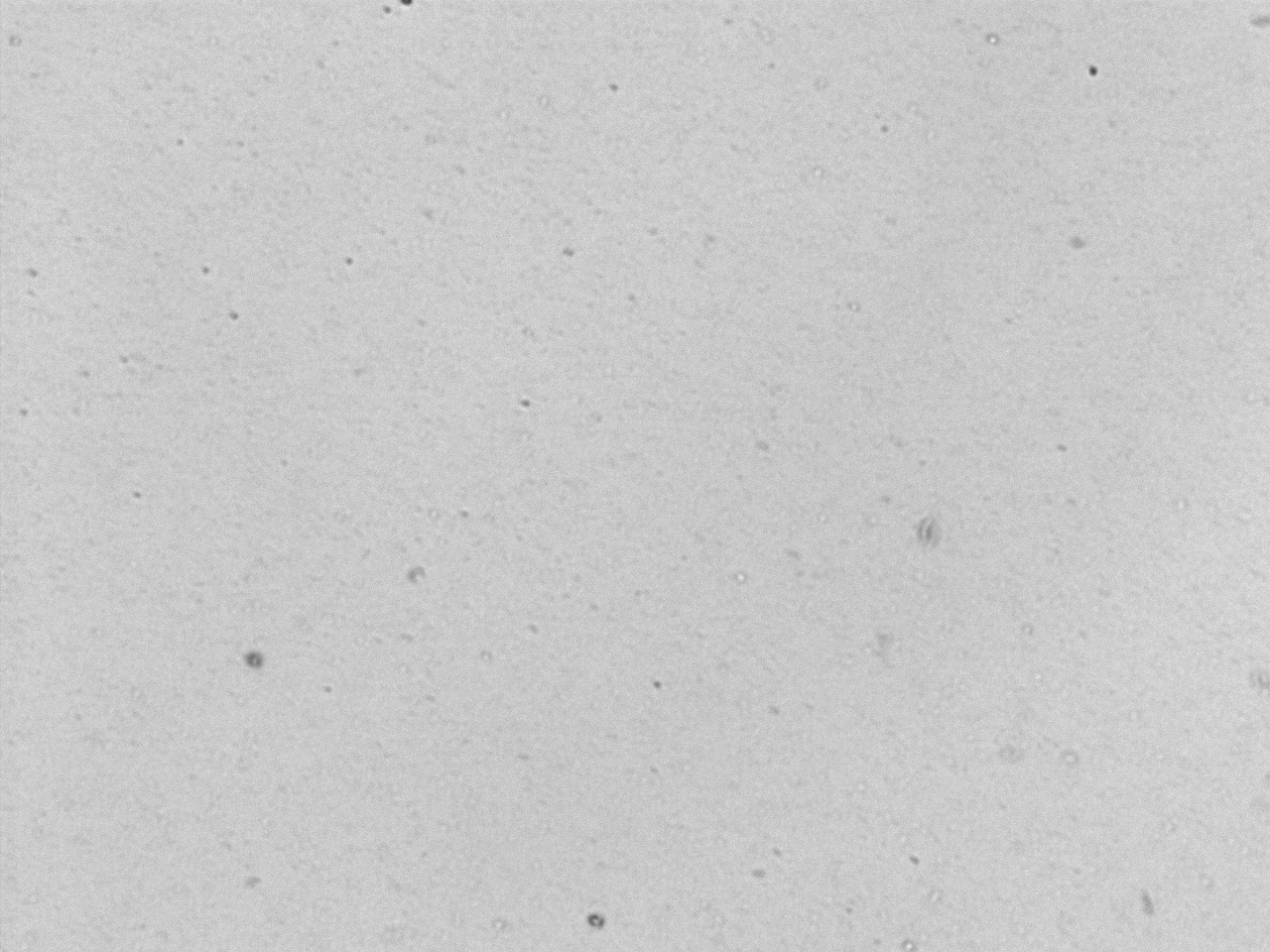

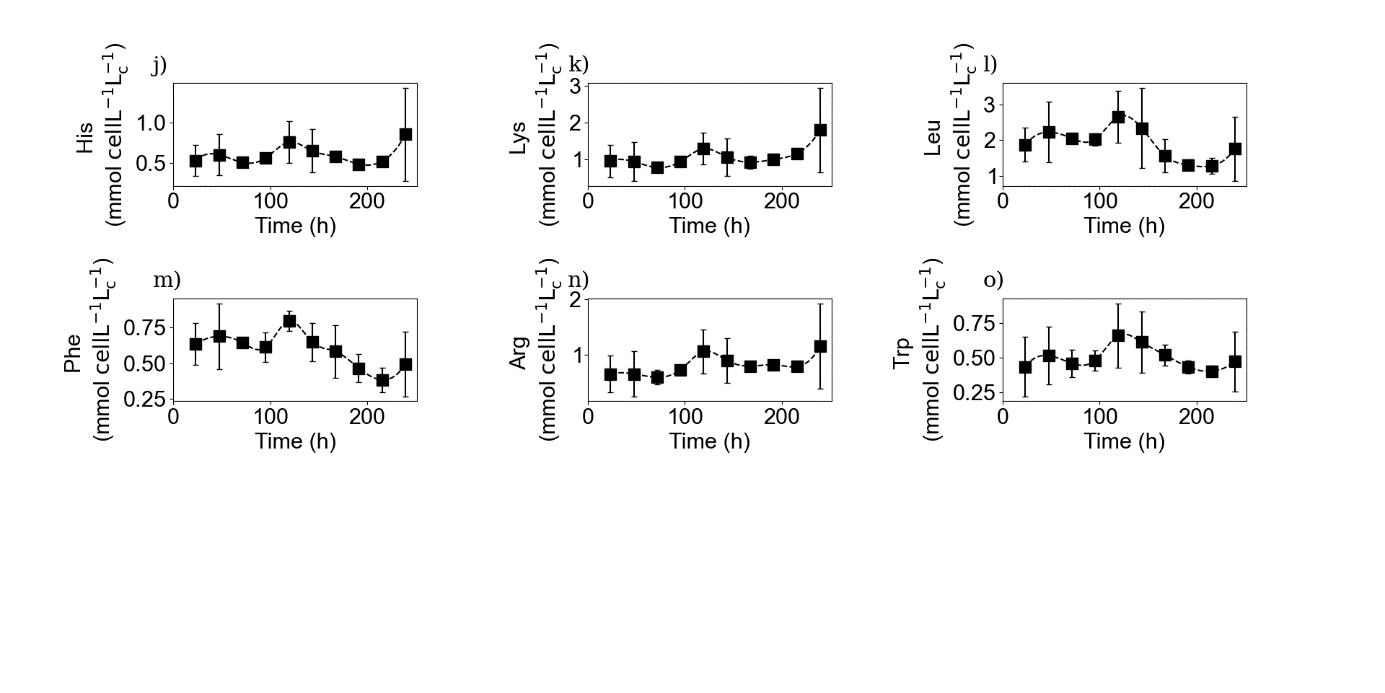

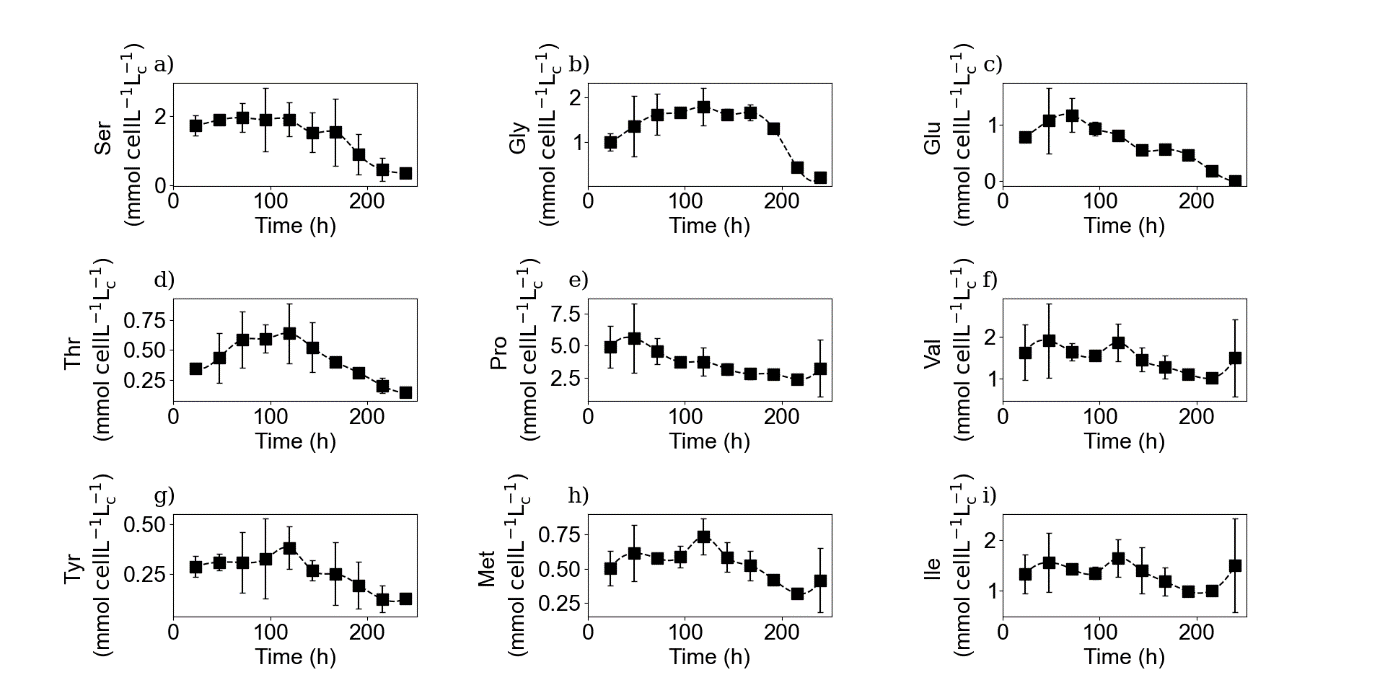


**Figure S 4.** Experimental intracellular amino acids concentrations. Squares represent the average of the measured concentrations from 2 repetitions (E1 and E3).


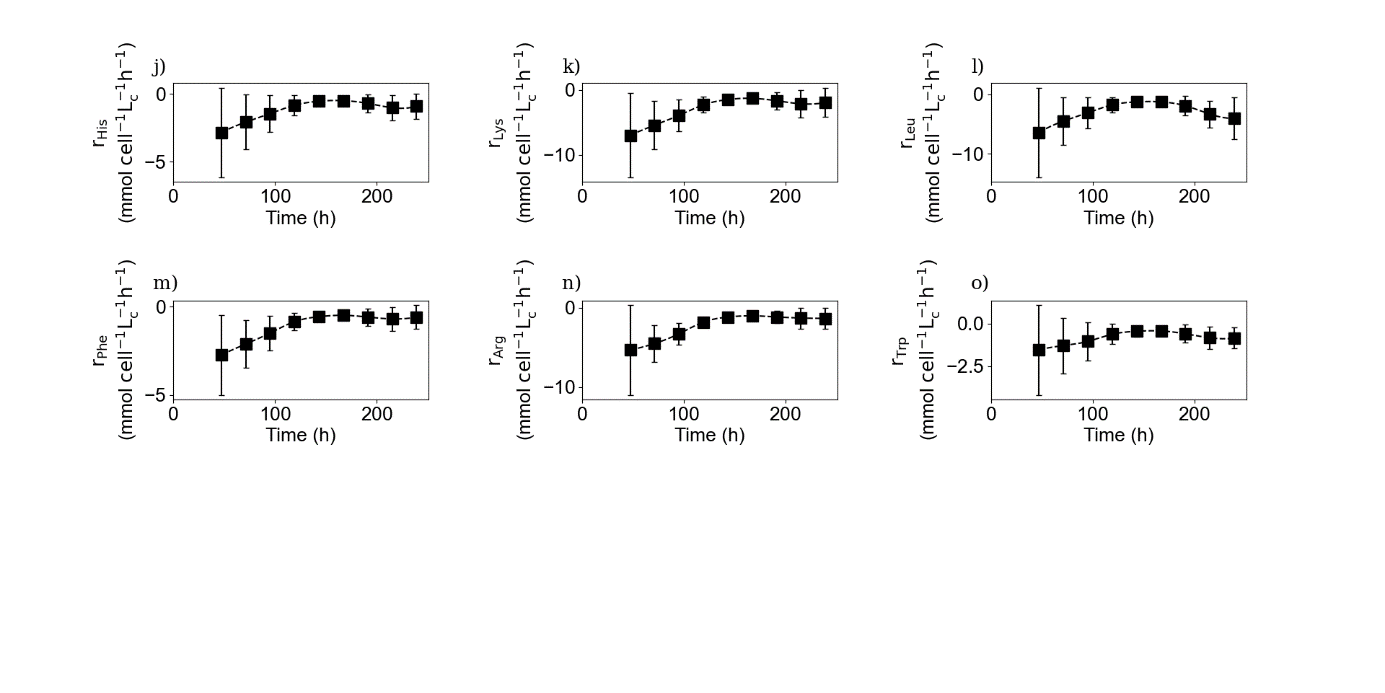

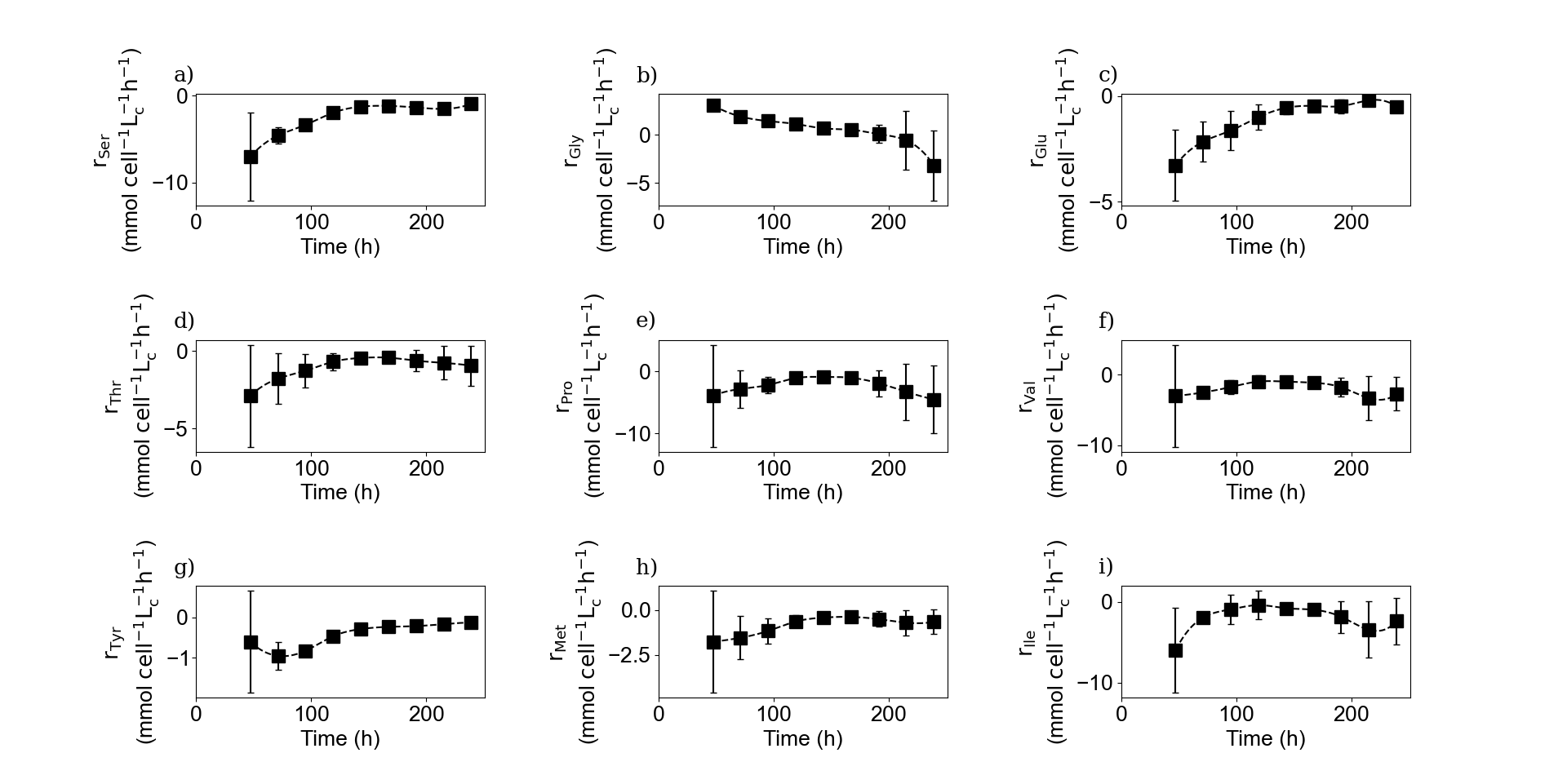


**Figure S 5**. Calculated intracellular amino acids reactions rates. Squares represent the average of the calculated reaction rates from 2 repetitions (E1 and E3).
